# Male terminalia of Cercopidae (Hemiptera, Cicadomorpha): towards a consensus terminology

**DOI:** 10.1038/s41598-021-89759-3

**Published:** 2021-05-17

**Authors:** Maxime Le Cesne, Elorde Crispolon, Adeline Soulier-Perkins

**Affiliations:** 1 Department of Entomology, College of Agriculture, University of Southern Mindanao, Kabacan Cotabato, Philippines; 2 Museum National d’Histoire Naturelle, Mécanismes Adaptatifs et Évolution (MECADEV): UMR 7179 MNHN-CNRS, CP 50, Entomologie, 45 rue Buffon, 75005 Paris, France

**Keywords:** Taxonomy, Entomology

## Abstract

The study of male genital appendages is often necessary to identify a species and to characterise the higher systematics ranks for the Cercopidae, a large family of Hemiptera. Therefore, many authors have used them in their work but without any clear consensus on the terms used for each part constituting the male terminalia. A standardised terminology is important for the quality of a taxonomic description but even more essential when we want to compare species and establish a primary homology between states of character and their use in the frame of phylogenetic analysis. The use of a consensus terminology should ensure that we are all observing, speaking and describing the same genital appendage and comparing homologous characters. In order to propose a consensus terminology, we have reviewed all the major works on the anatomy of terminalia for the family since the first description using those characters in 1922. We proposed the use of consensual terms, listed with their definitions. In addition we studied a diversified panel of male specimens, chosen in order to represent as many Cercopidae tribes as possible. We categorised five different groups of Cercopidae according to their male terminalia structures. This opens the reflection on the evolutionary patterns for these structures.

## Introduction

The Cercopidae constitutes a diverse family found around the world, with 1540 species described and distributed in 176 genera^1^. They are xylem-feeders feeding on a large range of plants. Adults are found above ground, feeding on different parts of the plants, while some of the nymphs sap suck on roots^2,3^.

The terminologies used to describe the different body parts of species in taxonomy can differ greatly depending on the group, authors and over time. The lack of accurate and precise terminology makes it impossible to compare characters rigorously. It is a problem not only for an accurate identification but for building a phylogenetic analysis and using characters for which primary homology should be evaluated and stated prior to comparing data described for the compared species. In 1970, Tuxen^4^ made a glossary referencing all the terms used through the different studies of the genitalia of insects with their definition. Nevertheless, discrepancy between the terminology and the specific organ applies, and remains in most groups, Cercopidae are no exception. Singh-Pruthi^5^ mentioned in his works on Rhynchota, that the works of several authorities proving the importance of genitalia in classification, show that in the absence of a detailed morphological account there has been a great multiplicity of terms and a considerable amount of confusion in their application.

The conservatism in the body shape and the convergence in the colouration patterns observed within the Cercopidae family make the identification to the generic or specific level challenging^6^. When working on South American Cercopidae, Fennah in 1968^7^ stated that female and male genital characters can be used for grouping species since they can be considered the least likely to have been influenced by parallel evolution. Male terminalia structures can also be diagnostic for species identifications, but in the majority of the old taxonomic literature, illustration and/or description of such diagnostic features is lacking, making the identifications more complicated^8^. However the use of genitalia characters is not new for this group^9–11^ but as predicted by Singh-Pruthi in 1925^12^, and shown by Fennah^7^, the male genitalia play a decisive role in the discrimination of the species. Therefore it is a necessary tool in every taxonomic work on Cercopidae. Numerous works on the morphology of Hemipteran genitalia have been done through the years^5,12–16^. Some tried to homogenize the terminology but no consensus has been reached yet and many different terms remain, used by authors and producing literature that can be tricky^3,6,17–27^. In this work, focusing on the Cercopidae, we propose to review the terminologies and to standardise it for the different pieces constituting the male terminalia.

## Cercopidae systematics

Suborder Cicadomorpha Evans, 1946^28^

Superfamily Cercopoidea Leach, 1815^29^

Family Cercopidae Leach, 1815^29^

Type species: *Cercopis sanguinolenta* (Scopoli, 1763)^30^.

In 1961^31^, the family Cercopidae was classically divided into two subfamilies: Cercopinae Leach, 1815^29^ and Callitettixinae Metcalf 1961^31^. In 1968, Fennah^7^ was working on Cercopidae from the New World and grouped them according to the structures of male and female genitalia. He suggested subdividing the family into two subfamilies, the Tomaspidinae containing all the New World genera and the Cercopinae dedicated to the Old World genera. He ignored the Callitettixinae because he was working on the New World taxa. According to Fennah, the Tomaspidinae generally present a metatibiae bearing two lateral spines, the male subgenital plates appear as an extension of the pygofer without any separation or groove and the first female valvulae bear some basal processes. In 2001, Hamilton^32^ described a new family from the American tropics, the Epipygidae and suggested including the Aphrophoridae as a subfamily in the Cercopidae. Cryan and Svenson in 2010^33^ presented the first phylogenetic molecular investigation on the Cercopoidea, which suggests that four of the five described families, Cercopidae, Clastopteridae, Machaerotidae and Epypigidae are monophyletic, the latest being nested within the Aphrophoridae. According to the selection of genera present in the phylogeny, a monophyletic lineage for the New World can be observed and designated as subfamily Ischnorhininae (sensu Carvalho & Webb 2005^6^). Paladini et al*.* in 2015^22^ follow Cryan & Svenson and propose to subdivide the subfamily in tree tribes: Ischnorhinini, Neanini and Tomaspidini. In 2018, Paladini et al*.*^34^ indicate a need to revise the genera included in the Tomaspidini and Ischnorhinini since those tribes do not remain monophyletic in their molecular analyses. According to Liang and Webb (2002)^3^, the current Old World classification of Cercopidae, together with many generic concepts, is based on the work of Lallemand^11^, using the number of spines on the hind tibia, and characters of the head, pronotum and fore wings. The Cercopinae is composed of numerous tribes and a series of *incertae sedis* genera. The monophyly of this subfamily was never tested. Liang and Webb^3^ revised the Rhinaulacini from southern Asia. It is the latest morphological work done at a tribal level for the Cercopinae. Cryan and Svenson^33^ point out that the taxonomic sample included in their phylogeny did not allow a comprehensive examination of Old World tribal structure, but some trends are emerging and suggest that their generic constituency needs to be examined in greater detail. If the construction of a molecular phylogeny for the Old World genera is needed in order to better understand their relationships, and the study of morphological structures should not be neglected. In order to be able to compare those structures we have to be sure that we compare the same structures. Male terminalia are no exception.

### Results and discussion on the general morphology of male terminalia (Fig. 1)

In 1910, Jacobi^9^ valued the inclusion of the male terminalia description and their illustration, through the description of a series of new species of Cercopidae. This practice is now widespread among the authors and became a necessity in the description of each taxon. In the meantime, this generated numerous terminologies. For the Hemiptera different scientists made some attempt to homogenise the vocabulary used in this group, such as Crampton 1922^13^, Singh-Pruthi 1925^12^, Snodgrass 1935^14^, Kramer 1950^15^, and Marks 1951^16^. As presented in Table 1, no consensus has been reached yet. The absence of standardisation in the terminologies can lead to misidentification, confusion in identification keys or homology recognition, difficulty in the communication between scientists, and inefficiency in scientific results (Bourgoin et al. 2015)^35^. If for some terms, such as pygofer, subgenital plates and aedeagus a consensus seems to have emerged, others are still described under different names. Finally, other structures were mentioned recently since their presence was observed only in few taxa. This is the case for the lateral and intermediate plates, respectively mentioned by Liang and Webb^3^ and Soulier-Perkins and Le Cesne^24^. We propose here a consensual terminology regarding the practices of authors in this domain (Table 1).

**Figure 1:**
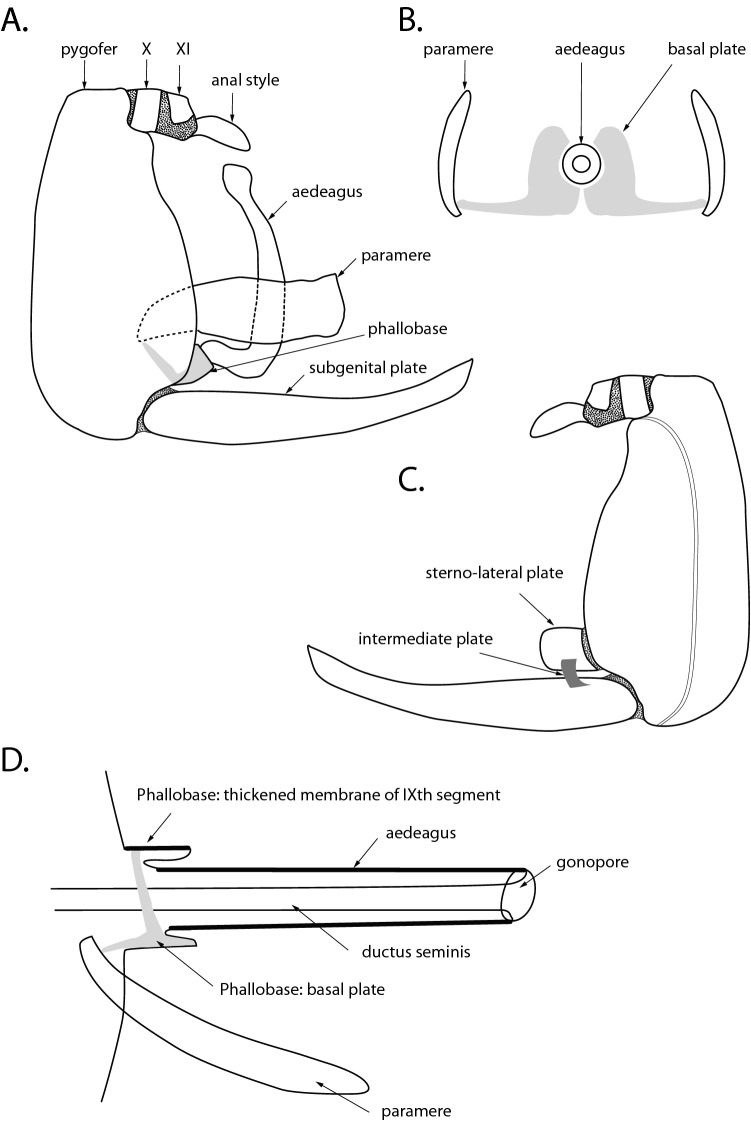
Schematic representation for the morphology of male terminalia. (**A**) Lateral view of a male terminalia. (**B**) Antero-posterior view of the parameres, basal plates and aedeagus. (**C**) Internal view of the right side of the Terminalia, showing the subgenital plate, the sterno-lateral plate and the intermediate plate in between. (**D**) Lateral view of the Aedeagus and phallobase.

**Table 1:** Terminologies used by different authors since Crampton, 1922 to designate different parts of the male terminalia.

Proposed terminology	Pygofer	Anal tube	Subgenital plates	Intermediate plates	Sterno-lateral plates	Parameres	Phallobase	Aedeagus
Crampton, 1922^13^	Ninth sternite or Hypandrium	–	Hypovalvae, Hypandrial valves	–	–	Styli, Gonostyli, Gonopods	**–**	Aedeagus
Singh-Pruthi, 1925^12^	Pygofer	**–**	Sub-genital plates	**–**	**–**	Parameres	Perandrium	Aedeagus
Snodgrass, 1935^14^	**–**	**–**	-	**–**	**–**	Harpagones	Phallobase	Aedeagus
Lallemand, 1949^11^	IXth segment	Anal tube	Genital plates	**–**	**–**	Styles	–	Penis
Kramer, 1950^15^	IXth segment	Anal tube	Subgenital plates	**–**	**–**	Parameres	Basal plates	Aedeagus
Marks, 1951^16^	IXth sternum	Anal tube	**–**	**–**	**–**	Gonoforceps	Basal plates	Aedeagus
Fennah, 1968^7^	Pygofer	Anal segment	Subgenital plates	**–**	**–**	Genital styles	**–**	Aedeagus
Hamilton and Morales, 1992^36^	Pygofer	Anal tube	Subgenital plates	**–**	**–**	Syles	Phallobase	Aedeagus
							Penis	
Yang and Chang, 2000	IXth segment	IX–X segments	Genital plates	–	–	Genital styles	Phallobase	Aedeagus
Liang and Webb, 2002^3^	Pygofer	Anal tube	Subgenital plates	–	Lateral plates	Styles	-	Aedeagus
Liang, 2003^18^	Pygofer	Anal tube	Subgenital plates	–	Basal plates	Genital styles	–	Aedeagus
Carvalho and Webb, 2005^6^	Pygofer	–	Subgenital plates	–	–	Parameres	Connective	Aedeagus
	Genital capsule							
Soulier-Perkins and Kunz, 2012^23^	Pygofer	Anal tube	Subgenital plates	–	Lateral plates	Parameres	–	Aedeagus
Liang et al. 2012^19^	Pygofer	Anal tube	Subgenital plates	–	Lateral plates	Genital styles	–	Aedeagus
Paladini et al. 2015^22^	Pygofer	Anal tube	Subgenital plates	–	-	Parameres	–	Aedeagus
Soulier-Perkins and Le Cesne, 2016^24^	Pygofer	Anal tube	Subgenital plates	Intermediate plates	Lateral plates	Parameres	–	Aedeagus

#### Terminalia

We group under this term the pygofer and the structures it bears plus the anal tube.

#### Pygofer (Fig. 1A)

Here we consider it as being the ninth abdominal segment. However certain authors introduce some subtlety. Sing-Pruthi^12^ refers to the pygofers as the large and conspicuous lateral regions of the ninth abdominal segment. Crampton^13^ refers to the ninth sternite when he uses the term hypandrium. In the Cercopidae, it is a ring-like structure composed of the tergite and sternite, excluding the subgenital plates appearing as an appendage of it.

#### Anal tube (Fig. 1A)

It corresponds to the tenth and eleventh segments. The sclerites of the tenth and eleventh segments are separated by inter-segmental membrane) and terminate in an elongated spoon-shaped process under the anus, the anal style as described by Singh-Pruthi^12^.

#### Subgenital plates (Fig. 1A)

They are a pair of plates arising from the posterior ventral margin of the pygofer as referred to by Hamilton and Morales^36^. Sing-Pruthi^12^ refers to them as a pair of well-developed appendages of the ninth sternite. They can be flexible but are never provided by muscles. In some groups, these appendages have developed in continuity with the pygofer and are fused to it. Some intermediate cases can be observed as well. The three alternatives can be observed for the Cercopidae.

#### Parameres (Fig. 1A,B)

Crampton calls them Styles, gonostyli or gonopods and mentions their connections with the base of the aedeagus by what he calls a connective. Singh-Pruthi^12^ lists the terms claspers, laterals or genital styles as synonyms. Snodgrass calls them harpagones and describes them as a paired structure articulated to some part of the ninth segment. Individually provided with muscles, they arise from the floor of the genital chamber and are connected to the sclerites of the phallobase via the connectives. These appendages are generally placed on each side of the aedeagus.

#### Aedeagus (Fig. 1A,B,D)

It is a median tubular structure^12^ unequally chitinised. Some authors call the aedeagus plus the phallobase, the penis, or phallus. When the theca of the phallobase is developed and completely or partially covers the aedeagus, they can be called the aedeagus *sensus lato*, the aedeaegus *sensus stricto* being only the aedeagus itself (Aed). In the case of the Cercopidae, the theca is completely reduced and the term aedeagus refers to the aedeagus *sensus stricto*. It communicates to the body cavity by the ejaculatory duct, which enters through the basal foramen^12^. This basal foramen is located in between the two basal sclerites, the basal plates, nested in the body wall or genital chamber. These sclerotic parts sometimes form a ring from which the aedeagus projects and provide attachments to phallic muscles. It is what we generally observe for the Cercopidae^14^. The whole aedeagus is sclerotized except for the most apical parts that seems to correspond to the endophallus. At the end, the gonopore opens.

#### Phallobase (Fig. 1A,B,D)

As defined by Tuxen^4^, it is the whole structure supporting the aedeagus. For the Cercopidae it is reduced to a small ring at the base of the aedeagus. It is composed of the basal plates (BsP), nested in the genital chamber, extending toward the parameres and the thickened segmental membrane of the IXth segment covering them. Tuxen also mentions the connective as being a sclerotized structure belonging to the phallobase and connecting it to the styles and mentions that for the Auchenorrhyncha, it is a synonym for basal plate. It is Snodgrass in 1935^14^, who describes the phallobase for the first time as being the proximal part of the phallus highly variable in its development, sometimes a large structure supporting the aedeagus, often produced in a thecal fold of sheath about the aedeagus, sometimes represented only by basal phallic sclerites in the wall of the genital chamber. Since then, in descriptive works, the sclerified basal parts,extending toward the parameres took different names. Yang and Chang in 2000^37^, call it basal part phallobase, when Carvalho and Webb^6^ call it connective.

#### Sterno-lateral plates (Fig. 1C)

They are a pair of plates present only in some genera of Cercopidae. First described by Liang and Webb^3^, they seem to have the same origin as the subgenital plates, they arise from the ninth sternite and are generally flexible but are not provided with muscles. They are located above the subgenital plates, on the pygofer margin.

#### Intermediate plates (ItP, Fig. 1C)

These structures are paired like the subgenital and lateral plates. They were observed first in 2016 by Soulier-Perkins and Le Cesne^24^ and illustrated as well in Crispolon et al*.* 2019^27^. When present, the intermediate plate links the subgenital plate to the lateral plate and often takes the shape of a small bridge.

All five families of Cercopoidea possess male genitalia with the same basic parts even if in the literature we encounter different terminologies. Using common terms to all of them should be an achievable goal. The terminology problems increase when we compare these structures to those described in other groups within the Auchenorrhyncha. The term pygofer is used in all the other families of Cercopoidea^38–44^, as well as in Cicadellidae^45,46^ and Membracidae^47–50^ but pygofer does not cover quite the same structures. In Membracoidea only the tergite is considered the pygofer. The sternite, which may be fused or articulated to the tergite, is called the valve, and the subgenital plates, which also may be either fused or articulated to the valve (IXth sternite) are usually not considered part of the pygofer. In the Fulgoromorpha the term pygofer includes tergite and sternite^14,51^ and there is no question about the subgenital plates that are absent in this group. In the Cercopoidea, because the tergite is fused to the sternite, it seems logical to call this ring like structure pygofer and consider the subgenital plates separately, like for the Membracoidea. Those subgenital plates are a pair of plates arising from the IXth sternite for all the cercopoids^38–44^, Cicadellidae^45,46^ and Membracidae^47–50^. The term lateral plate is used for the Cercopidae for the first time by Liang and Webb^3^ then Soulier-Perkins and Kunz^23^. These plates are present in some tribes of the old world Cercopidae, they arise like the subgenital plates from the IXth sternite. However, the term lateral plate is also used for the Membracidae^47–50^ and designates a synapomorphic character for this group and this distinctly delimited structure, either entirely or distally free or entirely fused to the pygofer, originating from the IXth tergite. So, if in both groups we consider that the lateral plates arise from the pygofer, they are not of the same origin, and are not of homologous structures. For these reasons, we suggest changing the term lateral plate to sterno-lateral plate for the Cercopidae in order to emphasise their different origin. The appendages on each side of the aedeagus are generally named parameres in Cercopidae^6,12,15,22,23^ and Machaerotidae^52^ when the term style is preferred for the Aphrophoridae^39–43^, Clastopteridae^42,44^, Epipygidae^38^ and Membracoidea^46–50^. Even though the term style is used for the Fulgoromorpha the term gonostyle is widely used as well^51,53^. Authors generally describe the aedeagus as the median tubular part bearing the ejaculatory duct. To refer to the sclerotized part the term theca is sometimes used, for instance for the Clastopteridae by Hamilton^42^ or aedeagal shaft for the Cercopidae^19,22^. At the base of the aedeagus, the reduced phallobase is essentially represented by a thickened membrane of the IXth segment and the basal plates, which support the ejaculatory duct and connect the phallic structure to the parameres. This structure is not always drawn and named in taxonomic works, but when it is represented it can be called connective^6,50^ or just labelled as phallobase^32,37,42^. For some Fulgoromorpha such as the Tropiduchidae, Bourgoin and Huang^51^ describe the phallobase *sensus lato* as being a synonym of the periandrium and what they describe as phallobase *sensus stricto* (sensus Fennah 1945^54^) is a development of this structure folding around the aedeagus in an external sclerified phalotheca and an internal membranous endotheca. This phallobase *sensus stricto* can be considered absent in the Cercopidae. They described what remains as a thickening of the diaphragm which corresponds to what we called thickened segmental membrane of the IXth segment. Bourgoin and Huang also describe precisely what they call the connective *sensus lato* an internal composite structure. The first part is composed at the base by a small pregenital invagination of the diaphragm called the ventral support, followed by the body of the connective, which runs in the general cavity and finishes in a gutter shape structure. This first part is the connective *sensus stricto* and it is topped by the second part, the tectiform structure. In between these two parts the ejaculatory duct goes through and rest in the gutter-shaped part. The tectiform structure does not exist in the Cercopoidea and the fulgoromorphan’ connective is difficult to synonymise with the term connective used for the Cercopoidae. In both cases the described structures link the parameres to the base of the phallus and support the ejaculatory duct but are not quite of the same origin.

Focusing on the Cercopidae, and according to the definitions provided above and the observations done on specimens selected to illustrate the cercopid diversity, we observe different degree of fusion of the subgenital plates to the pygofer, the presence or absence of sterno-lateral plates and when those plates are present another pair of plates can sometimes be observed as well, the intermediate plates.

Fennah in his work of 1968^7^ pointed out the importance of the genital characters to study the Cercopidae and considered them as not being influenced by parallel evolution. Without discussing the higher rank of the family, Fennah is the first to mention the different configuration of the male terminalia and that it could reflect the phylogenetic relationship of family. He expressed it by dividing the Cercopidae in two subfamilies but did not deepen it. His hypothesis was recovered by a first phylogeny of the Cercopidae of the world^33^, where the new world cercopids derived from the Old Word. They suggested that the genital characters reflect the same evolution. As such, a complete fusion of the subgenital plates with the pygofer is a derived state compared to a partially fused or distinctly separated subgenital plates to the pygofer. The presence of sterno-lateral plates is only observed in some genera found in the Old world and could be a derived state and a synapomorphy within a monophyletic lineage. The appearance of intermediate plates linking the sterno-lateral plate to the subgenital plate could be a step further within the evolution of this character, but those hypotheses remain mere speculation for now since the phylogeny of the whole Cercopidae family is not yet available. The optimisation of these patterns should be done when it becomes possible to test them. It has to be pointed at as well that when the subgenital plates are partially or completely fused to the pygofer, no sterno-lateral plates can be observed.

**Figure 2:**
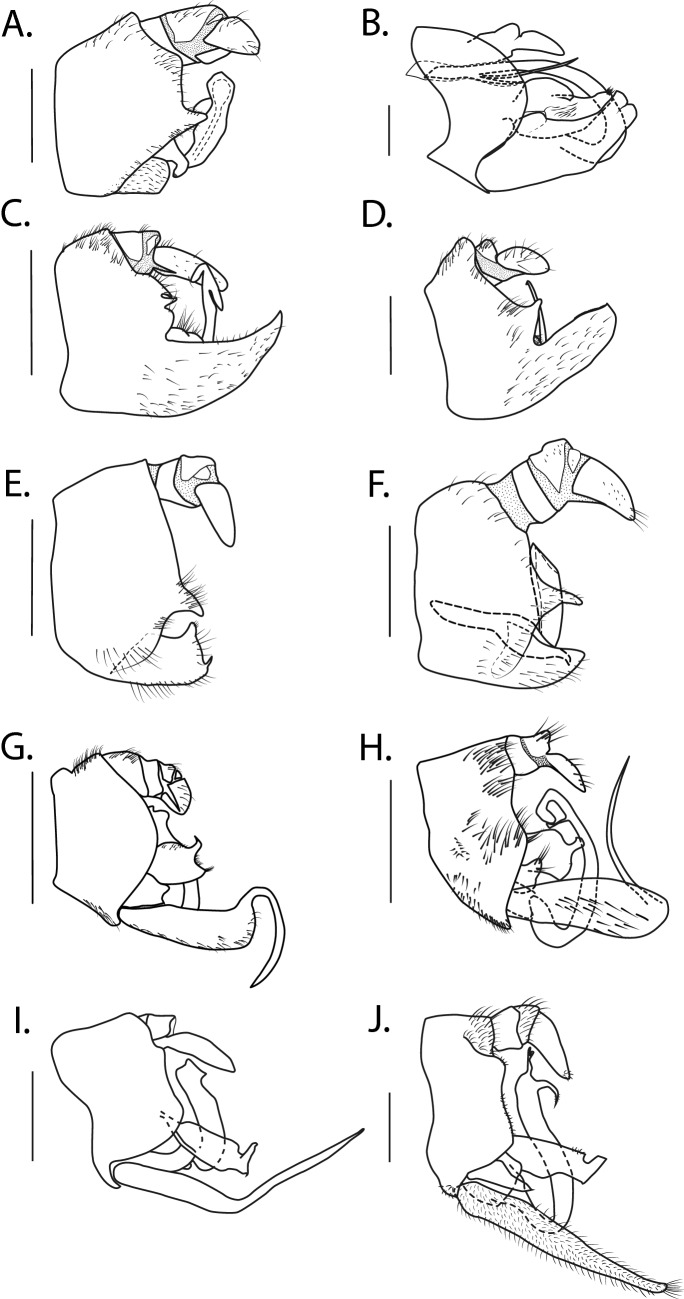
Different morphological configurations observed for the male terminalia. (**A**) *Considia nitidula* (Breddin, 1902), (**B**) *Cercopis sanguinolenta* (Scopoli, 1763), (**C**) *Pachacanthocnemis bella* (Walker, 1851), (**D**) *Prosapia simulans* (Walker, 1858), (**E**) *Cosmoscarta herossa* Jacobi 1921, (**F**) *Radioscarta* sp. (**G**) *Eoscarta borealis* (Distant, 1878) (**H**) *Callitettix versicolor* (Fabricius, 1794), (**I**) *Aufidus albonigrus* Le Cesne & Souliers 2021 *J. Wawi mehi* Soulier-Perkins & Cesne 2016.

For now, as a practical tool, but without any intention to describe different states of evolution, we propose to group the male terminalia of Cercopidae in five categories (Table 2). As illustration for the group 1, we can take the type-species, *Cercopis sanguinolenta* for which the subgenital plates are clearly distinct from the pygofer and do not possess any other plates, neither a sterno-lateral nor an intermediate plate (Fig. 2A,B). The group 2 includes all the New World cercopids observed. It is characterised by the fusion between the subgenital plates and the pygofer, the subgenital plates appear as an elongation of the sternite IX without any distinction from it (Fig. 2C,D). Those terminalia do not possess any additional plates. The group 3 possess subgenital plates partly fused to the pygofer, however a fold is visible, on each side, a groove is running down but does not reach the ventral part (Fig. 2E,F). If the pygofer is generally shaped as a uniform sclerotized ring, in this third group, a ventral narrow antero-posterior membranous band can be observed. The group 4 presents some subgenital plates clearly distinct from the pygofer, some extra structures can be observed, above the subgenital plates. Those structures are the sterno-lateral plates (Fig. 2G,H). A groove individualises them from the pygofer. The group 5, similar to group 4, possess a pair of subgenital plates distinct from the pygofer, a pair of sterno-lateral plates and a pair of intermediate plates that are, small sclerotised plates, shaped as a bridge and linking the subgenital plate to the sterno-lateral plate (Fig. 2I,J).

**Table 2:** Species studied with the subfamily, tribe, biogeographical region and terminalia group to which they belong.

Subfamilies	Tribes	Species	Biogeographic regions	Subgenital fused to the pygofer	Sterno-lateral plates present	Inetermediate plates present	Terminalia group
Calitettixinae	Callitettixini	*Abidama producta* (Walker, 1851)	Oriental	No	Yes	No	4
		*Callitettix versicolor* (Fabricius, 1794)	Oriental	No	Yes	No	4
	Considiini	*Considia nitidula* (Breddin, 1902)	Oriental	No	No	No	3
Cercopinae	Cercopini	*Cercopis sanguinolenta* (Scopoli, 1763)	Palearctic	No	No	No	1
		*Hemitriecphora haglundi* (Schouteden, 1901)	Afrotropical	No	No	No	1
	Cosmoscartini	*Cosmoscarta herossa* Jacobi, 1921	Oriental	Only partially	No	No	3
		*Ectomnnotum bivittatum* (Le Peletier de Saint-Fargeau & Serville, 1825)	Oriental	Only partially	No	No	3
	Euryaulacini	*Euryaulax carnifex* (Fabricius, 1775)	Oceanic	No	Yes	Yes	5
		*Leptataspis discolor* (Boisduval, 1835)	Oriental & Oceanic	Only partially	No	No	3
	Locridini	*Locris vicina* (Signoret, 1860)	Afrotropical	No	No	No	1
	Rhinaulacini	*Aufidus albonigrus* Le Cesne & Soulier-Perkins	Oceanic	No	Yes	Yes	5
		*Eoscarta borealis* (Distant, 1878)	Oriental & Oceanic	No	Yes	No	4
		*Mioscarta obscuripennis *Schmidt, 1920	Oriental	No	Yes	Yes	5
		*Paramioscarta brunnea* (Lallemand, 1920)	Afrotropical	No	Yes	No	4
		*Poeciloterpa mangkas* Crispolon & Yap, 2019	Oriental	No	Yes	Yes	5
		*Poeciloterpa minuta* Lallemand, 1922	Oriental	No	Yes	Yes	5
		*Wawi mehi* Soulier-Perkins & Le Cesne, 2016	Oceanic	No	Yes	Yes	5
	Trichoscartini	*Trichoscarta roborea* (Distant, 1900)	Oriental	Only partially	No	No	3
	Suracartini	*Opistharsostethus demonstratus* (Distant, 1900)	Oriental	Only partially	No	No	3
		*Simeliria viridans* (Guérin-Méneville, 1834)	Oriental	Only partially	No	No	3
		*Suracarta basinotata* (Butler, 1874)	Oriental	Only partially	No	No	3
Ischnorhininae	Ischnorhinini	*Laccogrypota valida* (Distant, 1909)	Neotropical	Yes	No	No	2
	Neaenini	*Zuata raviella* (Lallemand, 1924)	Neotropical	Yes	No	No	2
	Tomaspini	*Aeneolamia contigua* (Walker, 1851)	Neotropical	Yes	No	No	2
		*Huaina inca* (Guérin-Méneville, 1844)	Neotropical	Yes	No	No	2
		*Pacahcantocnemis bella* (Walker, 1851)	Neotropical	Yes	No	No	2
		*Prosapia simulans* (Walker, 1858)	Neotropical	Yes	No	No	2
*Incertae sedis*	*Incertae sedis*	*Phymatostetha* sp.	Oriental	No	No	No	1
		*Pogonorhinella madagascariensis* Schmidt, 1910	Afrotropical	No	No	No	1
		*Radioscarta* sp.	Oriental	Only partially	No	No	3

## Material and method

### Preparation

The abdomen of each specimen examined was cut off and cleared for 20 min in warm (80 °C) 10% KOH. Dissections and cleaning of genital structures were performed in distilled water. If needed, a few drops of blue paragon for dying the ectodermic genital ducts were added for a few minutes. Final observations were done in glycerine using a Leica microscope (MZ16).

### Abbreviations

IRScNB: Royal Belgian Institute of Natural Sciences.

MNHN: National Museum of Natural History, Paris.

NHM: British Museum, London.

MNH-UPLB: Museum of Natural History, University of the Philippines Los Baños, Philippines.

### Material examined

*Abidama producta *(Walker, 1851)^55^: 1 male, [Avril-Juin], [Museum Paris; Tonkin Central, Region de Tuyen-Quan et de Dong-Chau; A. Weiss, 1901], [Museum Paris; MNHN(EH) 24755].

*Aeneolamia contigua *(Walker, 1851)^55^: 1 male, [Museum Paris; Venezuela Llanos; F. Geay, 33–96], [Museum Paris; MNHN(EH) 24756].

*Aufidus albonigrus* Le Cesne & Soulier-Perkins, 2021^56^: 1 male, [Holotype], [Papua New Guinea, Madang province, Wanang 180 m, S5° 13′ 40″ E145° 04′ 47″], [Museum Paris, PL camp, 27-XI-2012, A. Soulier-Perkins rec.], [MNHN(EH)24057].

*Callitettix versicolor* (Fabricius, 1794)^57^: 1 male, [Vietnam, Kien Giang, Hon Chong, Kien Luong, Nui Bai Voi hospital-cave, 10°13.358’N 104°36.588’E], [Museum Paris; 4-VI-2008; calcareous hill, 17 m; A. Soulier-Perkins rec.], [Callitettix versicolor (Fabricius, 1794); M. Le Cesne det. 2019], [ Museum Paris; MNHN(EH) 24656].

*Cercopis sanguinolenta* (Scopoli, 1763)^30^: 1 male, [44° 57′ 0–03″ N, 5° 44′ 0–10″ E; La Motte d’Aveillans, Isère, France; 25.V.12, coll. by O. Béthoux], [sanguinolenta Scopoli, 1763; Cercopis sanguinolenta det. by O. Béthoux, 2012], [IWC OB 1046], [Cercopis sanguinolenta (Scopoli, 1763); A. Soulier-Perkins det. 2017], [Museum Paris; MNHN(EH) 24542].

*Considia nitidula* (Breddin, 1902)^58^: 1 male, [Museum Paris; Perak,Coll. Noualhier 1898], [$], [Considia nitidula (Breddin, 1902); M. Le Cesne det. 2020], [Museum Paris; MNHN(EH) 24737].

*Cosmoscarta herossa* Jacobi, 1921^59^: 1 male, [Tonkin; Cho-Ganh; L. Duport], [Collection E. Fleutiaux], [Cosmoscarta herossa Jacobi, 1921; M. Le Cesne det. 2020], [Museum Paris; MNHN(EH) 24658].

*Ectomnonotum bivittatum* (Le Peletier de Saint-Fargeau & Serville, 1825)^60^: 1 male, [Museum Paris; M^es^ du Ht Song-Chai; Rabier 258–95], [Ectemnonotum bivittatum var. flavisfascium Walk.], [Museum Paris; MNHN(EH) 24645].

*Eoscarta borealis* (Distant, 1878)^61^: 1 male, [Museum Paris; Tonkin, reg. de HOA-BINH; A DE COOMAN 1926], [Museum Paris; MNHN(EH) 24660].

*Euryaulax carnifex* (Fabricius, 1775)^62^: 1 male, [Australie; Chilagoe GPS300; 11/12-III-1997; Th. Bourgoin rec.], [Museum Paris; piège lumineux; Th. Bourgoin rec.], [Euryaulax carnifex (Fabricius, 1775); M. Le Cesne det. 2020], [Museum Paris; MNHN(EH) 24753].

*Hemitriecphora haglundi* (Schouteden, 1901)^63^: 1 male, [La Maboké; Rep. Cenrafric.; V.1966; Michel Boulard], [Muséum Paris], [Hemitriecphora haglundi (Schouteden, 1901); M. Le Cesne det. 2020], [Museum Paris; MNHN(EH) 24648].

*Huaina inca* (Guérin-Méneville, 1844)^64^: 1 male, [Mexique, Malinalco; Mexico; 24-X-1977; D. Pluot Rec.], [Museum Paris], [Huaina inca (Guérin-Méneville, 1844); M. Le Cesne det. 2020], [Museum Paris; MNHN(EH) 24624].

*Leptataspis discolor* (Boisduval, 1835)^65^: 1 male, [Museum Paris; I. Waigeoe; coll. Noahlhier, 1898], [Leptataspis discolor (Boisduval, 1835); M. Le Cesne det. 2020], [Museum Paris; MNHN(EH) 24650].

*Laccogrypota valida *(Distant, 1909)^66^: 1 male, [18/08/2009; Pocaré; Nouragues; Guyanne; piege vitres], [Museum Paris; MNHN(EH) 24662].

*Locris vicina* (Signoret, 1860)^67^: 1 male, [20/III/2006; 633 m; parc de Zombitse, Leobondro; brd rivière; 22° 40.460′ S 44° 51.633′ E], [Museum Paris; Madagascar 2006; reg. Atsimo-andrefana, A; Soulier-Perkins réc.], [Locris vicina (Signoret, 1860); A. Soulier-Perkins det. 2007], [$], [Museum Paris; MNHN(EH) 24659].

*Mioscarta obscuripennis *Schmidt, 1920^68^: 1 male, [Philippines, Negros; volcan Canlaon, forêt; N 10**°**25’. 31’’ E 123**°**05.43’’], [Museum Paris; piège lumineux, 1098 m asl; 30-X-2010; A Soulier-Perkins rec.], [Museum Paris; MNHN(EH) 24661].

*Opistharsostethus demonstratus* (Distant, 1900)^69^: 1 male, [Indonesia, Borneo; West Kalimantan prov.; Mount Bawang; 1400 m] [Museum Paris; VIII-2012; don Ph Magnien], [*Opistharsostethus demonstratus*], [Museum Paris; MNHN (EH) 24691].

*Pachacanthocnemis bella* (Walker, 1851)^55^: 1 male, [Museum Paris, EQUATEUR LOCA (Ex. Coll. A. DAVID, R. OBERTHUR 1903], [Museum Paris; MNHN(EH) 24847].

*Paramioscarta brunnea* (Lallemand, 1920)^70^: 1 male, [22/III/2006; Anjà, réserve village; forêt, 1000 m; 21° 51.120′ S 46° 50.773′ E], [Museum Paris; Madagascar 2006; rég. Haute Matsiatra; A. Soulier-Perkins réc.], [Paramioscarta brunnea (Lallemand, 1920); M. Le Cesne det. 2019], [Museum Paris, MNHN(EH) 24657].

*Phymatostetha* sp.: 1 male, [Forestry P.I.; 100 m; IV-28–58; P. P. Bandong], [UPLBMNH; HEM-04214]. (MNH-UPLB).

*Poeciloterpa mangkas* Crispolon & Yap 2019^27^: Male Holotype, [Philippines, Negros; volcan Canlaon, forêt; N 10**° **25′ 31′′ E 123**° **05.40′′], [Museum Paris; piège lumineux; 1098 m asl; 29-X-2010; A Soulier-Perkins rec.], [Museum Paris; MNHN(EH) 23642].

*Poeciloterpa minuta* Lallemand 1922^71^: Male paratype, [Mt. Makiling; Luzon, Baker], [Lallemand Coll. B.M.1955-832], [Paratype]. (NHM).

*Pogonorhinella madagascariensis* Schmidt, 1910^72^: 1 male, [Madagascar; province of Toamasina; Andasibe, 1049 m; S18° 53,410′ E48° 23,881′], [Museum Paris; forêt humide, brd piste nickel; 05-XI-2011; A. Soulier-Perkins Rec.], [Pogonorhinella madagascariensis Schmidt, 1910; M. Le Cesne det. 2019], [Museum Paris, MNHN(EH) 24119].

*Prosapia simulans* (Walker, 1858)^73^: 1 male, [Mexiq] [Museum Paris, Coll. G. Fallou 259-95] [*Tomaspis simulans* Walker] [*Prosapia simulans* (Walker, 1858), M. Le Cesne det. 2016], Museum Paris; [MNHN(EH) 24848].

*Radioscarta *sp.: 1 male, [Coll. I.R.Sc.N.B.; Singapore Nee Soon Mal Trap 2; Station 25091; 27-IV-05; swamp forest; Leg P. Grootaert]. (IRScNB).

*Simeliria viridans* (Guérin-Méneville, 1834)^74^: 1 male, [Museum Paris; Java, Tjibogo, Preanger (J B Ledru); R. Oberthur, 1898], [*Simeliria viridans*], [Museum Paris; MNHN (EH) 24692].

*Suracarta basinotata* (Butler, 1874)^75^: 1 male, [Suracarta sp ?; Michel Boulard det. 19.], [90-24]s, [Suracarta tricolor var basinotata (Butl.); det. A.P. Liang ‘90], [Museum Paris; MNHN(EH) 24649].

*Trichoscarta roborea* (Distant, 1900)^69^: 1 male, [Museum Paris; Borneo; R. Oberthur, 1898], [Museum Paris; MNHN(EH) 24754].

*Wawi mehi* Soulier-Perkins & Le Cesne, 2016^24^: Male holotype, [Papoua-New-Guinea; Mt Wilhelm; 2073 m; S5° 45′ 32′′ E145° 11′ 10′′], [Museum Paris; Malaise trap; 27-X-2012], [Museum Paris; MNHN(EH) 22754], [Wawi mehi Soulier-Perkins & Le Cesne, 2016; A. Soulier-Perkins det. 2016].

*Zuata ravidella* (Lallemand, 1924)^76^: 1 male, [Museum Paris; Bolivia; Coll. Noualhier, 1898], [Museum Paris; MNHN(EH) 24693].
